# Tumor-Associated Macrophages in Hepatocellular Carcinoma Pathogenesis, Prognosis and Therapy

**DOI:** 10.3390/cancers14010226

**Published:** 2022-01-04

**Authors:** Konstantinos Arvanitakis, Triantafyllia Koletsa, Ioannis Mitroulis, Georgios Germanidis

**Affiliations:** 1 First Department of Internal Medicine, AHEPA University Hospital, Aristotle University of Thessaloniki, 54636 Thessaloniki, Greece; kostarvanit@gmail.com; 2 Basic and Translational Research Unit, Special Unit for Biomedical Research and Education, School of Medicine, Faculty of Health Sciences, Aristotle University of Thessaloniki, 54636 Thessaloniki, Greece; 3 Department of Pathology, School of Medicine, Aristotle University of Thessaloniki, 54124 Thessaloniki, Greece; tkoletsa@auth.gr; 4 First Department of Internal Medicine, University Hospital of Alexandroupolis, Democritus University of Thrace, 68100 Alexandroupolis, Greece

**Keywords:** tumor-associated macrophages, hepatocellular carcinoma, tumorigenesis, tumor microenvironment, treatment resistance

## Abstract

**Simple Summary:**

Hepatocellular carcinoma (HCC) constitutes a major health burden, accounting for >80% of primary liver cancers globally. Inflammation has come into the spotlight as a hallmark of cancer, and it is evident that tumor-associated inflammation drives the involvement of monocytes in tumor growth and metastasis. Tumor-associated macrophages (TAMs) actively participate in tumor-related inflammation, representing the main type of inflammatory cells in the tumor microenvironment, setting the crosstalk between tumor and stromal cells. Infiltrating TAMs exert either anti-tumorigenic (M1) or pro-tumorigenic (M2) functions. In most solid human tumors, increased TAM infiltration has been associated with enhanced tumor growth and metastasis, while other studies showcase that under certain conditions, TAMs exhibit cytotoxic and tumoricidal activity, inhibiting the progression of cancer. In this review, we summarize the current evidence on the role of macrophages in the pathogenesis and progression of HCC and we highlight their potential utilization in HCC prognosis and therapy.

**Abstract:**

Hepatocellular carcinoma (HCC) constitutes a major health burden globally, and it is caused by intrinsic genetic mutations acting in concert with a multitude of epigenetic and extrinsic risk factors. Cancer induces myelopoiesis in the bone marrow, as well as the mobilization of hematopoietic stem and progenitor cells, which reside in the spleen. Monocytes produced in the bone marrow and the spleen further infiltrate tumors, where they differentiate into tumor-associated macrophages (TAMs). The relationship between chronic inflammation and hepatocarcinogenesis has been thoroughly investigated over the past decade; however, several aspects of the role of TAMs in HCC development are yet to be determined. In response to certain stimuli and signaling, monocytes differentiate into macrophages with antitumor properties, which are classified as M1-like. On the other hand, under different stimuli and signaling, the polarization of macrophages shifts towards an M2-like phenotype with a tumor promoting capacity. M2-like macrophages drive tumor growth both directly and indirectly, via the suppression of cytotoxic cell populations, including CD8+ T cells and NK cells. The tumor microenvironment affects the response to immunotherapies. Therefore, an enhanced understanding of its immunobiology is essential for the development of next-generation immunotherapies. The utilization of various monocyte-centered anticancer treatment modalities has been under clinical investigation, selectively targeting and modulating the processes of monocyte recruitment, activation and migration. This review summarizes the current evidence on the role of TAMs in HCC pathogenesis and progression, as well as in their potential involvement in tumor therapy, shedding light on emerging anticancer treatment methods targeting monocytes.

## 1. Introduction

Liver cancer is the sixth most commonly diagnosed cancer and the fourth leading cause of cancer-related death worldwide, while hepatocellular carcinoma (HCC) accounts for approximately 75–85% of primary liver cancers [1,2]. In the vast majority of cases, HCC arises as a result of sustained inflammatory damage, hepatocyte necrosis and regeneration in patients with liver cirrhosis [3]. The most common causes of liver cirrhosis that predispose patients to HCC are chronic hepatitis C virus (HCV), hepatitis B virus (HBV) and non-alcoholic fatty liver disease/non-alcoholic steatohepatitis (NAFLD/NASH) [4]. Despite major scientific advances, the majority of patients with HCC still face a dismal prognosis. Liver transplantation is only possible in patients with early HCC, whereas patients that undergo alternative treatment modalities—including liver resection or tumor ablation—develop recurrent disease in up to 70% of cases [5]. HCC prognosis fluctuates according to the stage at the time of diagnosis, with an overall 5-year survival rate of 20%. Patients who are not considered eligible for surgical or other curative procedures can receive palliative therapies, including tyrosine kinase inhibitors (TKI), such as sorafenib, lenvatinib, regorafenib or cabozantinib, or the VEGFR2-antibody ramucirumab [6,7,8,9]. However, these treatment options have minimal benefit in survival, increasing the necessity for novel therapeutic strategies for the treatment of HCC [10]. Recent evidence provided by Finn et al. showed that the use of the immune checkpoint inhibitor atezolizumab in combination with the antiangiogenic agent bevacizumab reduced mortality by 42% and decreased the risk of disease worsening or death by 41% compared to sorafenib alone, and it is currently accepted as the first-line systemic treatment of HCC [11]. Immunotherapies targeting the PD-1/PD-L1 axis, other than atezolizumab, have also been approved for the treatment of HCC without showing a major effect on patient survival [12]. Specifically, the efficacy of these agents is compromised in patients with NASH and this observation was linked to NASH-dependent altered immune cell function in TME [13].

Chronic liver inflammation drives a dysfunctional tissue repair process, leading to the formation of dysplastic nodules and, eventually, cancer [14,15]. The tumor microenvironment (TME) greatly contributes to the tolerogenic immune response towards HCC. It comprises myeloid-derived suppressor cells (MDSCs), tumor-associated macrophages (TAMs), tumor-associated neutrophils (TANs), cancer-associated fibroblasts (CAFs) and regulatory T cells (Tregs) [16,17,18]. MDSCs, either of monocytic or granulocytic origin, typically show immunosuppressive properties [19]. The liver bears the highest proportion of macrophages among all organs in the body [20], and in a healthy rodent liver, 20–40 macrophages accompany every 100 hepatocytes [21]. They are generally categorized into two distinct subsets that can be distinguished from each other based on their differential expression of cell surface markers. Kupffer cells (KCs) are the non-migratory tissue-resident macrophages of the liver, are located in the sinusoids and maintain homeostasis [22,23]. Monocyte-derived macrophages (MoMϕs) exert migratory capabilities and engraft liver tissue during inflammatory conditions or after KC depletion [24,25]. TAMs play an essential role in HCC pathogenesis, establishing a pro-inflammatory and pro-tumorigenic environment through the suppression of antitumor immune responses [26,27]. However, they can also participate in tumor immune surveillance and antitumor responses [28,29]. Given the major contribution of hepatic macrophages in normal tissue homeostasis, their pivotal role in liver inflammation and their dual promoting and inhibitory functions in tumor formation, hepatic macrophages have been at the forefront as potential therapeutic targets for various HCC treatment modalities [30].

This review summarizes the current state of knowledge regarding the involvement of TAMs in the pathogenesis and progression of HCC and the heterogeneity and plasticity that they exert in the cancer-associated microenvironment. We also highlight the principal future therapeutic options that target TAMs to treat HCC.

## 2. Monopoiesis and Tumor-Associated Monocytes

One of the main features of cancer is the tumor-associated chronic inflammation that results in the reprogramming of immune cells [31]. In addition to the immunomodulatory effect that this type of chronic inflammation elicits at the site of tumors, it has systemic effects, affecting cell populations in the bone marrow and spleen. One of these effects is the induction of emergency myelopoiesis, which results in the generation of mature cells of the myeloid lineage, including monocytes and neutrophils, that further infiltrate solid tumors and act mainly as immunosuppressive and tumor-promoting cells [32]. Hematopoietic stem and progenitor cells (HSPCs) and myeloid progenitors (MyP) can sense and are responsive to a variety of mediators that are released by tumor cells, such as granulocyte-colony stimulating factor (G-CSF), granulocyte macrophage (GM)-CSF or the chemokine CXCL-12 [33,34]. This results in their activation and differentiation towards a myeloid lineage, as well as in their mobilization and egress from the bone marrow and migration to extra medullary sites, such as the spleen [35].

**Figure 1:**
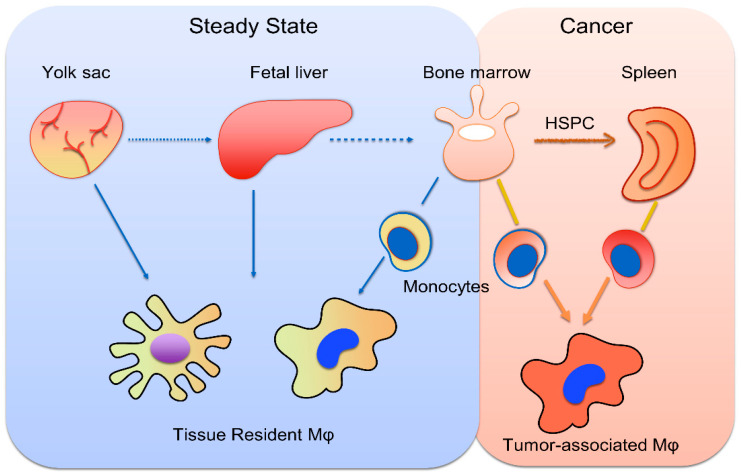
Origins of tissue-resident and tumor-associated macrophages. Tissue-resident macrophages derive from the differentiation of yolk sac and fetal liver hematopoietic progenitors and, later in life, stem from the differentiation of monocytes, generated in the bone marrow. Cancer induces myelopoiesis in the bone marrow, as well as the mobilization of hematopoietic stem cells and progenitor cells (HSPCs), which reside in the spleen. Monocytes produced in the bone marrow and the spleen further infiltrate tumors, where they differentiate into tumor-associated macrophages.

Resident macrophages derive from the differentiation of progenitors in the yolk sack and fetal liver during early life, whereas bone marrow is the site of monocyte generation under steady state conditions [36]. Experiments in mice showed that monocytes derive from common myeloid progenitors, following two distinct differentiation pathways, giving rise to cells with different transcriptional programs and, probably, different functions [37]. Cancer-elicited inflammation promotes not only the generation of monocytes in the bone marrow in response to the myeloid-lineage growth factors GM-CSF, G-CSF and interleukin-6 (IL-6) [33], but also drives extramedullary monopoiesis in the spleen by mobilized HSPCs and MyP [38]. Indeed, both mice and patients with invasive cancer exhibited increased numbers of splenic granulocyte-macrophage progenitors (GMPs), which are able to generate monocytes, as well as neutrophils, that further infiltrate tumors and differentiate into TAMs and TANs, respectively [38]. The spleen is also a major site for the tumor-associated reprogramming of monocytes, which results in an accumulation of MDSCs, a monocytic cell population with potent immunosuppressive activity against CD8+ T cells [39]. For instance, a study by Jordan et al. showed that the accumulation of MDSCs of monocytic origin with T-cell-suppressive properties were observed in the spleens of patients with various types of cancer, including pancreatic and colorectal adenocarcinoma, pancreatic neuroendocrine tumors, melanoma or ovarian cancer [40]. Regarding HCC, increased numbers of hematopoietic progenitor cells, stained as CD133+, and CD11b+ myeloid cells were observed in the spleens of patients with HCC, as well as other tumors, compared to patients with cirrhosis, a condition that predisposes patients to HCC [41]. Interestingly, there was a positive correlation between the number of progenitor cells and mature myeloid cells, implying that extra medullary myelopoiesis was responsible for the generation of mature myeloid cells [41]. Taken together, enhanced myelopoiesis results in the generation of monocytic cells with immunosuppressive properties in the bone marrow and the spleen, which further migrate into tumors, where an additional step of reprogramming takes place during their differentiation to TAMs (Figure 1).

## 3. Liver Macrophages and Their Plasticity in Response to the Tumor Microenvironment

Liver macrophages (Mϕs) are a heterogeneous cell population that includes resident Kupffer cells and MoMϕs. On histological examination, Kupffer cells share common morphological characteristics with the monocyte-derived cells, MoMϕs and dendritic cells, showing morphological variability in size and shape. Kupffer cells are identified on light microscopy by their location, as they are localized adjacent to sinusoids and play a crucial role in homeostasis. MoMϕs are observed in inflammatory sites, orchestrating the immune response to tissue injury or pathogens. Mϕs play a major role in the pathogenesis of inflammatory disorders, such as NASH, promoting liver inflammation and fibrosis [42,43], as discussed in elsewhere [43,44].

The microenvironment of HCC, which consists of CAFs, hepatic stellate cells (HSCs), endothelial cells and immune cells, as well as extracellular matrix proteins [45], shapes Mϕs, altering their function. TAMs actively participate in tumor-related inflammation, setting the crosstalk between tumor and stromal cells [46,47]. TAMs are MoMϕs that are recruited into the TME, mostly by chemokine (C-C motif) ligand 2 (CCL2) and macrophage (M)-CSF, and eventually differentiate into mature macrophages. The plasticity of TAMs enables them to exert either anti- or pro-tumor activity, depending on the distinct micro-environmental signals originating from tumor cells, fibroblasts, stroma and immunocompetent cells [48]. TAMs show similar morphological features to their normal counterparts, despite their structural and functional diversity, which is controlled by the tumor microenvironment [49]. Similar to liver Mϕs, TAMs are functionally heterogeneous. However, in most human studies, TAMs are characterized based on the expression of polarization markers as classically activated (M1) and alternatively activated (M2). Classically activated M1 macrophages have pro-inflammatory activity and macrophages polarize to this direction in response to treatment with lipopolysaccharides (LPS) and interferon-gamma (IFN-γ). M1 macrophages produce proinflammatory cytokines, such as interleukin (IL)-12, and have the potential to stimulate effector T-cell proliferation and function. They also exhibit strong microbicidal and tumoricidal activity by the production of reactive oxygen species (ROS) and nitric oxide synthase (iNOS; NOS2) that promotes arginine metabolism into nitric oxide (NO) and citrulline [50,51]. Alternative activation by IL4, IL-10 and IL-13 in vitro results in the generation of macrophages with immunosuppressive properties that produce IL-10, transforming growth factor beta (TGF-β) and chemokine (C-C motif) ligand (CCL) family members, such as CCL17, CCL18, CCL22 and CCL24, and express high levels of PD-L1 [27,52]. M2 macrophages initiate the Th2 immune response, promoting angiogenesis, tissue remodeling and repair [53,54].

Histologically, the distinction between M1 and M2 macrophages is based on immunohistochemistry. CD68 is a common monocytic marker expressed by TAMs, of both M1 and M2 phenotypes, and dendritic cells. M1 macrophages are positive for several markers, such as iNOS, CD80 and CD86, whereas the M2 polarization markers are CD163, CD204 and CD206 [55,56]. CD163, in addition to being a marker for M2 macrophages, is also expressed by Kupffer cells. Moreover, a population of tolerogenic dendritic cells has been recently identified in peripheral blood, namely DC-10, which expresses CD163, releases IL-10 and induces type-1 T regulatory cells [57]. CD163 expression is upregulated by IL-10, and CD163-positive cells secrete IL-10.

The TME is the driving force behind macrophage polarization in HCC, leading to the generation of macrophages with immunosuppressive properties. For instance, the TME is characterized by an acidic pH, triggering regulatory macrophages and enhancing immune evasion [58]. Yang et al. demonstrated that Wnt/β-catenin signaling drives macrophage differentiation towards the M2 phenotype and was highly expressed in c-Myc-driven M2-polarized macrophages. Inhibition of Wnt protein secretion in HCC hindered hepatic tumor growth by regulating the tumor immune microenvironment in mice, whereas nuclear accumulation of β-catenin was observed in M2-like TAMs in human HCC biopsies [59]. Moreover, Chen et al. demonstrated that TLR4-elicited innate monocyte inflammation was necessary for IL21+ T follicular helper (Tfh)-like cell induction in HCC, and activation of STAT1 and STAT3 was critical for TFH-like cell polarization. Importantly, the TFH-like cells operated in IL21-IFNγ-dependent pathways to induce plasma cell differentiation and, thereby, create conditions for pro-tumorigenic M2 macrophage polarization and cancer progression [60]. Similarly, intratumoral macrophages were associated with increased intratumoral FoxP3+ regulatory T cells (Tregs) and poor prognosis in patients with HCC, while in vivo depletion of tissue macrophages decreased the frequency of intratumoral immunosuppressive FoxP3+ Tregs [61]. To underline the multitude and the complementarity of the mechanisms of local HCC immunosuppression, it was recently deduced that selectively increased intrahepatic Tregs can promote an immunosuppressive environment in NASH livers. Neutrophil extracellular traps link innate and adaptive immunity by promoting Treg differentiation via the metabolic reprogramming of naïve CD4+ T-cells. This mechanism may explain, at least partly, the relative resistance of NASH-related HCC to current first-line immunotherapies and could be targeted to prevent or treat liver cancer in patients with NASH etiology [62]. On the other hand, the extracellular matrix protein SPON2 and its integrin receptors α4β1 play significant roles in the recruitment of the M1-like TAM subtype in the HCC microenvironment, functioning as an opsonin for macrophage phagocytosis, resulting in anti-tumor immune responses [63]. Additionally, expression of MiR206 by Kupffer cells drives M1 polarization and the recruitment of CD8+ T cells through CCL2 production in mice with HCC [64]. In addition to T cells, Liu et al. reported that TAMs interact with B cells, since CXCR3+ B cells drive M2 polarization in HCC through IL-17 production [65]. The IL-6/STAT3 signaling pathway was shown to regulate M1/M2 macrophage polarization, as its inhibition, mediated by anti-IL6, reduced cell viability and drug resistance, suppressed cell invasion and migration and induced apoptosis of HCC cells co-cultured with M1- or M2-type macrophages, resulting in suppressed tumor formation and lung metastases [66]. In addition, TLR4 on macrophages promoted the growth of steatohepatitis-related HCC in mice, as the number of macrophages expressing Ly6C was increased and these cells were associated with inflammation and tumor progression, generating increased amounts of IL-6 and TNFα in response to LPS [67]. On the contrary, IL-12 inhibited HCC proliferation and invasiveness in vitro by the induction of M1-like polarization of macrophages through the downregulation of Stat-3 [68]. Furthermore, a study by Wang et al. demonstrated that TGF-β recruits M2 macrophages in HCC, which are in turn polarized by connective tissue growth factor (CTGF, CCN2), a protein expressed by mesenchymal-like HCC cells. TGF-β acts as a chemoattractant, recruiting monocytes from the peripheral blood, while CTGF acts as a transformant, polarizing monocytes to M2 macrophages, stimulating tumor growth. In turn, M2 macrophages secrete CCL18, promoting HCC cell migration [69]. Finally, the transition of the macrophage phenotype from antitumorigenic to protumorigenic, which has been proven to be mediated by c-Jun N-terminal phosphorylation in the liver microenvironment, occurs before overt tumorigenesis, resulting mostly in the production of CCL17 and CCL22, thus facilitating HCC growth [70,71].

**Figure 2:**
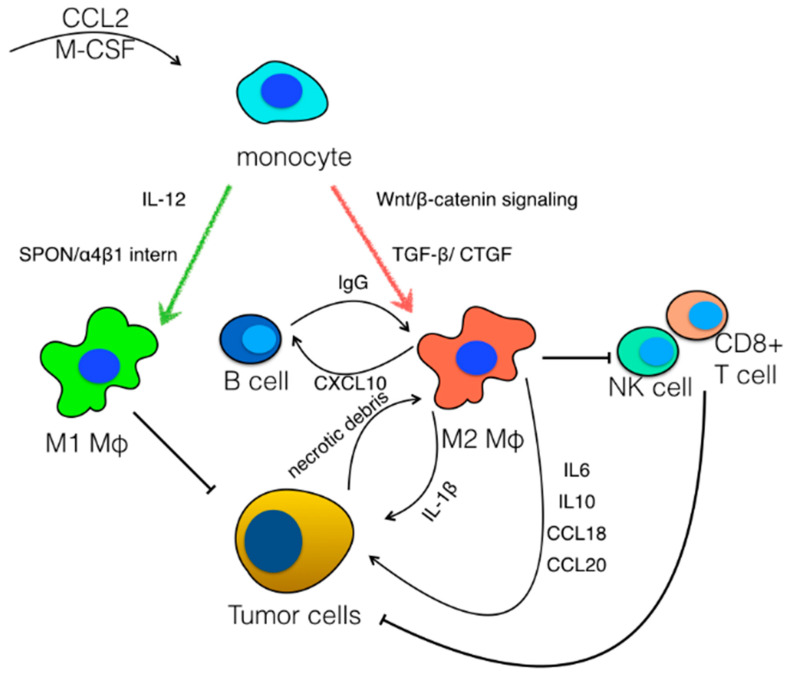
Polarization of tumor-associated macrophages. Macrophage colony-stimulating factor (M-CSF) and C–C motif chemokine ligand 2 (CCL2) drive the generation of monocytes in cancer. In response to IL-12 and SPON/α4β1 signaling, monocytes differentiate into macrophages with antitumor properties, which are classified as M1-like. On the other hand, Wnt/β-catenin signaling, the TGF-β/CTGF pathway, necrotic debris from tumor cells and immunoglobulins released by B cells facilitate the polarization of macrophages towards a M2-like phenotype with tumor promoting properties. M2-like macrophages drive tumor growth directly, through the release of IL-1β, IL-6, IL-10, CCL18 and CCL20, and indirectly, via the suppression of cytotoxic cell populations, including CD8+ T cells and NK cells.

Even though the aforementioned studies suggest that TAMs can be polarized between the two extremes of macrophage phenotypes, recent studies using single cell approaches demonstrated that TAMs in HCC are characterized by vast heterogeneity. A seminal study by Zhang et al. generated a large body of information regarding immune cell populations in HCC and ascites using single cell RNAseq of CD45+ cells [72]. Among others, six clusters of macrophages with distinct gene expression modules were identified [72]. Interestingly, they identified a cluster of macrophages that simultaneously expressed genes of both M1 and M2 polarization states [72]. Song et al. also engaged a similar experimental approach in HBV/HCV-related hepatocellular carcinoma and identified eight clusters of myeloid cells, showing that there is heterogeneity within macrophage populations, with a high number of macrophages sharing both M1 and M2 characteristics [73]. Among the different macrophage clusters, a cluster of CCL18-expressing macrophages with M2 features was identified that was associated with a worse clinical outcome [73]. In the same study, a population of XCL1^+^ CD8^+^ T cells was identified that was capable of recruiting dendritic cells, which resulted in an enhanced anti-tumor response, suggesting an interaction between T cells and myeloid cells [73]. In addition to the heterogenicity at the single cell level, it has been shown that immune cell infiltrates are distinct in intrahepatic metastatic lesions in multifocal HCC compared to multicentric occurrence, since more M2 macrophages and less T cells are observed in metastases [74] (Figure 2).

## 4. The Role of Macrophages in HCC Pathogenesis

The involvement of macrophages in the pathogenesis and development of HCC is crucial. Zhang et al. found that M2 macrophages increased the proliferation, migration and invasion of HCC cells through a fatty acid oxidation (FAO)-dependent process. Specifically, IL-1β instigated the pro-migratory effect of M2 cells, and FAO was responsible for the upregulated secretion of IL-1β, which depended on ROS and NLRP3 inflammasome [75]. A study on the diethylnitrosamine (DEN)-induced model of carcinogenesis engaged in transcriptomic analysis and demonstrated that MoMϕs acquire a proinflammatory phenotype during carcinogenesis in this model, which is, however, distinct from the mixed pro-inflammatory and immunosuppressive phenotype of cells from the NASH-induced carcinogenesis model [76]. This observation implies that there are diverse mechanisms in the response of immune cells to liver carcinogenesis in different animal models. Schneider et al. also demonstrated that DEN-induced hepatocarcinogenesis triggers liver inflammation with an intrahepatic accumulation of macrophages and cytotoxic T cells. Interestingly, the increased macrophage accumulation in chemokine scavenger receptor D6-deficient mice did not have an impact on HCC progression [77]. TAMs have also been linked to HCC growth stimulation via STAT3 signaling, while IL-6 release by macrophages was demonstrated to enhance HCC proliferation and migration [78]. Furthermore, evidence has been provided that chemokine CCL2/chemokine receptor CCR2-dependent signaling mechanisms participate in the process of HCC development. The levels of CCL2 are increased in patients with HCC and have been associated with poor prognosis [79]. In murine liver cancer models, CCR2+ myeloid cells exhibited dual functions. Before cancer initiation, CCR2+ myeloid cells suppressed tumorigenesis by removing senescent hepatocytes. However, when HCC was established, tumor cells inhibited the differentiation of infiltrating CCR2+ immature myeloid cells, which in turn promoted tumor growth, via the inhibition of NK cells. In a model of NASH-dependent HCC, CCR2 depletion had no distinct effect on HCC tumorigenesis, suggesting that the effect of CCR2 in hepatocarcinogenesis is dependent on disease etiology [80]. Guo et al. also provided evidence that infiltrating M2-TAMs were markedly elevated in the HCC TME, producing IL-17, a pro-inflammatory cytokine, and were augmented upon oxaliplatin treatment [81]. IL-17A, secreted in concert from lymphatic endothelial cells, promotes tumorigenesis by upregulation of PD-L1 in hepatoma stem cells [82]. Regarding HCC metastasis, it was demonstrated that the exosome-mediated transfer of the functional protein CD11b/CD18 (integrin αMβ2) from TAMs to tumor cells may have the potency to boost the migratory potential of HCC cells [83].

In addition, inhibition of the CCL2/CCR2 axis resulted in the blockade of monocyte recruitment, M2 polarization and, as a result, inhibition of the CD8+ T-cell-mediated antitumor response [84]. Zhang et al. demonstrated that the hypoxia inducible factor (HIF)-1α /IL-1β feedback loop between tumor cells and TAMs in the hypoxic TME resulted in the epithelial–mesenchymal transition of cancer cells and metastasis in vitro. Specifically, they found that TAMs secreted increased amounts of IL-1β under moderate hypoxic conditions, due to the increased stability of HIF-1α, and that the necrotic debris of HCC cells increased IL-1β release by TAMs with an M2 phenotype, via TLR4/TRIF/NF-κB signaling [85]. Moreover, Zhao et al. observed a positive association between B7-H1+ monocyte/Mφ and IL-17-producing cell density in the peritumoral stroma of HCC patients and that the IL-17-exposed macrophages suppressed cytotoxic T-cell function through B7-H1/PD-1 interactions [86]. CD48/2B4 interactions mediated a high level of infiltration of peritumoral macrophages, which was correlated with the decreased activity of NK cells in HCC tissues [87]. Furthermore, IL-23 generation by liver CD14+ inflammatory macrophages in response to infected hepatocytes during chronic HBV was shown to alter macrophage function, favoring HCC growth [88] (Table 1).

## 5. Macrophages in HCC Prognosis

The prognostic role of the proportion of M1 and M2 macrophages, as well as their ratio, have been reported in several tumors [91,92], including HCC [93]. M2 macrophages are implicated in the “exhausted immune response” subclass of HCC and are correlated with adverse prognosis [93,94]. The method for their quantification differs and is not comparable between studies. Density estimation, as reflected by the number/mm^2^, seems to be the most reliable method. Histologic distribution of TAMs and their subtypes, in the center of the tumor or the invasive front, has been proposed to have an independent prognostic value in several solid tumors [95,96]. Τhe histological evaluation of the invasive front is preferable to be performed in surgical specimens. Further research related to macrophage phenotypes and their ratios, in conjunction with their spatial location in the tumor specimens, can facilitate a better understanding of their biological behavior in HCC.

Recent evidence suggests that miR-148b deficiency promotes HCC growth and metastasis through colony-stimulating factor-1 (M-CSF)/CSF1 receptor (CSF1R)-mediated TAM infiltration, while HCC patients with decreased miR-148b levels and increased TAM infiltration were correlated with worse prognosis [97]. Moreover, Chen et al. reported that monocytes engage in glycolysis at the peritumoral region of human HCC, inducing PD-L1 expression and attenuating cytotoxic T lymphocyte responses in cancer. Tumor-derived soluble factors upregulated PFKFB3 expression in TAMs, which in turn mediated the increased expression of PD-L1 by the activation of the NF-kB signaling pathway. Interestingly, the degree of CD68 + PFKFB3 + PD-L1 + monocyte-macrophage infiltration in peritumoral tissues was negatively associated with the overall survival of HCC patients and could serve as an independent prognostic factor for patients with HCC [98]. In addition, Li et al. demonstrated that the downregulation of SIRT4 was correlated with increased macrophage infiltration and M2-like TAMs in HCC peritumoral tissues and, consequently, with poor survival of HCC patients. SIRT4 expression was decreased in macrophages in HCC, driving M2 polarization in a FAO-PPARδ-STAT3-dependent signaling pathway, while silencing SIRT4 increased IL-6 production in TAMs. Moreover, SIRT4 silencing also resulted in M1 macrophage apoptosis due to enhanced IL-10 production in HCC peritumoral tissues [99]. Along the same line, high-mobility group protein box1 (HMGB1) expression, which is linked to increased secretion of IL-1β, IFN-γ and TNF-α, was associated with peritumoral TAM infiltration and poor prognosis in patients with HCC. High peritumoral HMGB1 expression and TAM numbers were positively correlated with tumor size and BCLC stage and acted as independent prognostic factors for the overall survival (OS) and recurrence free survival (RFS) in patients with HCC [100]. Furthermore, Zhao et al. provided evidence that the expression of macrophage migration inhibitory factor (MIF) in tumors was positively correlated with plasma MIF levels, which had a higher value for the diagnosis of HCC compared to serum a-fetoprotein (AFP). In fact, plasma MIF levels demonstrated a significant correlation with the OS and disease-free survival (DFS) of HCC patients, even in those with normal serum AFP levels and tumor-node-metastasis (TNM) stage I. In addition, the plasma MIF levels were identified as an independent factor for OS and DFS and decreased significantly within 30 days after HCC resection [101]. Another study further reported that M-CSF density and the CD163 and CD31 indices in peritumoral tissues were predictable factors for time to recurrence, DFS and OS in patients with HCC, while M-CSF was involved in the progression of hepatocellular carcinoma after curative resection [102]. Moreover, hepatocellular tumors with increased intratumoral CD204, as well as monocarboxylate transporter-4 (MCT4)-positive macrophages and MCT4-postive expressing HCC cells, were associated with an unfavorable patient outcome [103].

Another study by Zhu et al. revealed that CD206 was highly expressed in the HCC tissues compared to its peri-carcinoma tissue levels, while GdCl3 treatment suppressed the malignant potential of HCC in vitro and in vivo, mainly by downregulating the expression of CD206 in M2 macrophages, indicating the potential significance of CD206 as a biomarker for HCC prognosis [104]. Similarly, high expression of peritumoral M-CSF and the density of macrophages, which correlated with a large tumor size, presence of intrahepatic metastasis and a high TNM stage, were associated with HCC progression, disease recurrence and poor survival after curative hepatectomy [105], while the combination of tumor-derived osteopontin (OPN) and peritumoral infiltrating macrophages was associated with a high incidence of early recurrence and poor survival for early-stage HCC, after curative resection [106]. A study by Zhou et al. demonstrated that Yes-Associated Protein (YAP) activation was critical for the recruitment of TAMs towards HCC cells, as IL-6 secreted by YAP-activated HCC cells induced TAM recruitment. Together with their findings that the expression levels of IL-6 in human HCC tumors were highly associated with the prognosis of HCC patients, they highlighted the possibility of improving HCC treatment by targeting YAP-IL-6-mediated TAM recruitment [107]. In addition, evidence has been provided that macrophages contribute to the decreased expression of E-cadherin in HCC via the NF–κB/Slug pathway, leading to increased tumor invasiveness and metastasis [108] (Table 2).

## 6. The Potential Role of Macrophages in HCC Treatment

The implication of macrophages in various HCC treatment modalities has been at the forefront of clinical investigation, as various preclinical studies in animal models suggest that macrophages can play a pivotal role in HCC therapy. For instance, macrophage depletion by clodrolip or zoledronic acid, in combination with sorafenib, significantly inhibited HCC progression, tumor angiogenesis and lung metastasis in mice [109]. In addition, sorafenib administration at a subpharmacologic dose, augmented the antitumor effects of mouse chimeric antigen receptor CAR-T cells, partly by promoting IL12 secretion from TAMs [110]. Along the same line, there is evidence that sorafenib triggers the proinflammatory activity of TAMs, reverts their alternative polarization, enhancing IL12 secretion, and, as a result, induces antitumor NK cell responses in a cytokine- and NF-kB-dependent fashion [111]. This ability of sorafenib to partially inhibit M2-cell activation in vivo was shown by a study by Sprinzl et al. [112]. Another study by Yao et al. indicated that a natural CCR2 antagonist potentiated TAM-mediated tumor immunosuppression and enhanced the therapeutic effect of sorafenib, indicating that the combination of an immunomodulator with a chemotherapeutic drug could be a therapeutic approach for HCC [113]. Macrophage modulation could also potentiate the anti-cancer activity of sorafenib. In addition, it has been shown that sorafenib inhibits the macrophage-mediated epithelial–mesenchymal transition in HCC via the HGF-Met signaling pathway in vitro, while sorafenib therapy reduced plasma HGF and alpha-fetoprotein concentrations in patients with HCC [114]. Finally, it inhibited miR-101 expression and enhanced DUSP1 expression and downregulated the release of TGF-β and the expression of CD206 in M2 cells, suppressing the macrophage-mediated growth of HCC [115].

A study by Yang et al. identified that 17β-estradiol (E2) could suppress HCC growth via regulation of macrophage polarization, as E2 re-administration reduced tumor growth in orthotopic and ectopic mouse HCC models, functioning as an inhibitor of macrophage alternative activation and tumor progression, by keeping estrogen receptor-β (ERβ) away from interacting with ATP5J and hindering the JAK1-STAT6 signaling pathway [116]. Moreover, delivery of recombinant adenovirus vector (rAd) expressing monocyte chemoattractant protein-1 (MCP-1) was demonstrated to potentiate the antitumor effects of suicide gene therapy against HCC by M1 macrophage activation, suggesting its potential use as a method of cancer gene therapy against HCC progression and recurrence [117]. Guerra et al. also demonstrated that, in response to HCC cells, hydrogel-embedded M1 macrophages upregulated nitrite and TNF-α, activating caspase-3-induced apoptosis in the tumor cells, leading to tumor regression in vivo [118]. It is also worth mentioning that small interfering RNA (siRNA)-mediated knockdown of MIF suppressed cyclin D1 expression and HCC cell proliferation, inducing tumor-cell apoptosis [119], while antibody mediated therapy targeting CD47 inhibited HCC progression, promoting the migration of macrophages into the tumor mass and the subsequent phagocytosis of HCC cells [120]. Tan et al. provided evidence that IRE1α mediated the inhibition of TAM activation by genipin in HCC, suppressing its growth, while the reduced association of IRE1α-TRAF2-IKK might have been responsible for a genipin-regulated inactivation of NF-κB [121]. Furthermore, co-administration of glycyrrhizin and doxorubicin by alginate nanogel particles was demonstrated to diminish the activation of macrophages through the regulation of the apoptosis pathway, via altering the Bax/Bcl-2 ratio and caspase-3 activity, enhancing the therapeutic efficacy for HCC [122]. Moreover, nanoliposome C6-ceramide (LipC6) was demonstrated to enhance the anti-tumor immune response and hinder HCC growth in mice, reducing the number of TAMs and their ability to suppress the anti-tumor immune response, allowing LipC6, a potential chemotherapeutic agent, to increase the efficacy of immune therapy in patients with HCC [123]. An additional in vivo study provided evidence that the strategy of low doses and multiple treatments of nsPEF was superior to a high dose of a single treatment, as macrophage infiltration was markedly elevated in tumors that were treated by multiple low dose nsPEFs [124]. Finally, nanosecond pulsed electric field (nsPEF), a technology targeting tumor cells with a non-thermal high-voltage electric field using ultra-short pulses, increased HCC cell phagocytosis by human macrophage cells (THP1) in vitro [125] (Table 3).

Recent advances in tissue engineering research enabled the construction of three-dimensional (3D) in vitro tissue models, in order to recapitulate the TME without engaging in vivo animal models. In these 3D culture systems, different types of cancer cells and cells that form the TME, including CAFs and TAMs, are combined providing valuable tools for the discovery of new drugs [126,127]. This type of experimental approach has already been used in a model of lung carcinoma to test whether treatment can alter TAM density and spatial distribution and it revealed that the treatment had an important effect on the latter [128]. Regarding HCC, hydrogels loaded with M1-polarized macrophages had a tumor suppressing potential both in vitro and in vivo [118].

## 7. Critical Analysis of Data and Future Perspectives

The landscape and dynamics of macrophages have been studied alongside other cell populations in human HCC multiple tissue compartments using single cell-RNA sequencing analysis [72]. It was identified that the enrichment of TAM gene signatures was significantly associated with a survival disadvantage in HCC, rendering this type of tumor-infiltrating TAMs potential cellular candidates for therapeutic targeting in the TME. Importantly, two genes, SLC40A1, encoding ferroportin, and GPNMB, encoding type I transmembrane glycoprotein, were highly expressed as potential markers in these TAM-like cells. Furthermore, in a second human study of an early HCC relapse ecosystem, a different innate-like CD8^+^ T cell population was described by single-cell profiling in recurrent tumors. These T cells were overexpressing the CD161 surface marker and displaying an innate-like low cytotoxic state, with low clonal expansion, unlike the classical CD8^+^ T cell exhausted state observed in primary HCC. The selective relative enrichment of these cells in the TME was associated with a worse prognosis in patients [129]. These unique aspects of altered immune response associated with HCC relapse relative to the primary tumor underline the HCC immune micro-ecosystem complexity of heterogeneous spatiotemporal interactions between and within cell types, which may guide the development of rational precision oncology immune therapies, benefiting a wide range of patients.

In accordance with the above, the HCC microenvironment in human patients and mice is characterized by functionally distinct macrophage populations. There are four possible main interventions for TAM-based antitumor therapy: inhibition of macrophage recruitment, induction of TAM death or apoptosis, enhancement of M1 antitumoral activity of TAMs and, last but not least, inhibition of the functional axes of M2 tumor-promoting activity of TAMs. In addition, functional subsets of TAMs were analyzed in human HCC samples and, in a combined fibrosis–HCC mouse model, demonstrated that human CCR2+ TAMs accumulated at the highly vascularized HCC border and expressed the inflammatory marker S100A9, whereas a second subset of CD163+ immune-suppressive TAMs accumulated in the HCC epicenter. Inhibition of CCR2+ TAM infiltration using a CCL2 antagonist in the fibrosis–HCC model significantly reduced pathogenic angiogenesis alongside tumor growth [90]. Moreover, the dual CCR2/CCR5 inhibitor cenicriviroc is currently under phase 3 clinical trial evaluation in patients with NASH and advanced fibrosis, representing a high-risk group for liver cancer [130]. However, the exact role of CCR2 and CCR5 in macrophage function in the liver is rather obscure. Recently, it was demonstrated that both CCR2 and CCR5 deficiency/inhibition led to reduced fibrosis, and sole CCR5 deficiency increased steatosis and the incidence of HCC in the model of NEMO LPC-KO mice. While CCR2 controlled the recruitment of monocytes to injured livers, CCR5+ macrophages limited liver injury in NEMOLPC-KO mice (CCR5-dependent differential function), thereby reducing steatosis and hepatocarcinogenesis [131]. In the hypoxic environment of HCC, HIF-1a enhanced the expression of triggering receptor expressed on myeloid cells-1 (TREM-1) in TAMs, leading to immunosuppression through the impairment of the cytotoxic functions of CD8+ T cells. Mechanistically, TREM-1+ TAMs increased the expression of CCL20 via the extracellular signal-regulated kinase/NF-κβ pathway in response to hypoxia and tumor metabolites leading to CCR6+ Foxp3+ Treg accumulation. Inhibition of the TREM-1 pathway could hinder tumor progression, reduce CCR6+ Foxp3+ Treg recruitment and improve the therapeutic efficacy of PD-L1 blockade [132].

It has been also indicated that the function of CCR2+ myeloid cells depends on the developmental stage of liver tumors. Precancerous senescent hepatocytes produce CCL2, which attracts macrophages, eliminating precancerous lesions (antitumoral effect), while established HCCs can also attract monocytic macrophages, which can, in turn, block the antitumor activity of NK cells (tumor-promoting effect) [79]. It has also been shown that selective blocking of CCR5 induces antitumoral macrophage polarization, and anti-CCR5 therapy was reported to be efficient in treating metastases [133]. Therefore, a critical review of the aforementioned data indicates that not all patients with HCC might eventually benefit from a selective CCR2- or CCR5-directed axis-inhibiting tumor therapy, while combined CCR2 and CCR5 inhibition is only beneficial for certain subgroups of patients with HCC. Due to the complicated nature of myeloid inflammation, multiple target inhibition might be necessary in order to overcome myeloid-mediated immune suppression. In this context, it was found that GM-CSF- and TNFa-producing CD206+ macrophages accumulated in human fibrotic liver. GM-CSF potentiated monocytes to CD206+ macrophage conversion, while anti-GM-CSF therapy suppressed liver fibrosis and CD206+ macrophage accumulation [134]. Furthermore, it was identified that tumor-derived GM-CSF was the primary regulator of myeloid cell ARG1 expression and local immune suppression. STAT3, p38 mitogen-activated protein kinases and acid signaling through cAMP were required to activate myeloid cell ARG1 expression in a STAT6-independent manner. A blockade of the tumor-derived GM-CSF enhanced the efficacy of tumor-specific adaptive T-cell therapy and immune checkpoint blockade [135]. Taken together, it seems that either monocyte or tumor cell–derived GM-CSF significantly contributed to the development of the immunosuppressive TME by regulating myeloid cell ARG1 expression and could serve as a target in order to enhance the efficacy of cancer immunotherapy.

There are currently several clinical trials investigating the use of monoclonal antibodies to inhibit GM-CSF or GM-CSFR in patients with various diseases. In a completed phase IIb study, a 24-week treatment with mavrilimumab, a human monoclonal antibody targeting the GM-CSFR a-chain, significantly reduced rheumatoid arthritis disease activity compared to placebo [136]. Moreover, GSK3196165, a human monoclonal antibody inhibiting GM-CSF, has also shown evidence of rapid favorable clinical responses in a phase Ib/IIa trial of patients with moderate RA [137]. In addition, it was demonstrated that GM-CSF neutralization with lenzilumab results in the reduction of neuro-inflammation and cytokine release syndrome in a primary acute lymphoblastic leukemia patient-derived xenograft model following chimeric antigen receptor T-cell therapy [138]. Furthermore, a phase ΙΙΙ trial is underway, investigating the potential use of lenzilumab to improve the likelihood of ventilator-free survival beyond standard supportive care, in hospitalized patients with severe SARS-CoV-2 [139]. Along the same line, recent evidence from studies of human and transplant mouse melanomas implicate CSF1 induction as a CD8+ T-cell–dependent adaptive resistance mechanism and demonstrate that simultaneous CSF1R targeting might be beneficial in melanomas refractory to immune checkpoint blockade and potentially, in other T-cell–based therapies [140]. Future findings from those ongoing trials and studies could provide insight into the potential use of GM-CSF-targeted therapies for the treatment of patients with HCC. Finally, subsets of tumor-associated innate immune cells, macrophages and neutrophils in particular, suppress the cytotoxic activity of innate and adaptive immune cells and interact with tumor cells to promote tumor growth and metastasis, suggesting that selectively targeting these sub-populations of TAMs and TANs holds therapeutic promise in treating metastatic disease [141].

## 8. Conclusions

Monocytes are highly adaptive cells that are influenced by the cytokine-chemokine milieu and are subsequently transformed by signals encountered upon entry into a tissue niche. Due to myeloid cell complexity and diversity, we are still far from understanding the complete set of internal and external signals that are sufficient to establish any particular monocyte-macrophage phenotype in the TME. Given the pro-metastatic role of monocytes in HCC, these shifts seem to have functional outcomes influencing disease state, rather than being simply epiphenomenal markers of the tumor and systemic environment.

Broadening our knowledge on how different signaling pathways regarding the recruitment and differentiation of monocytes interact with lineage-determining transcription factors, and how these factors interact with the overlaid differentiation factors within blood and tissue in cancer, might potentially enable us to intervene and define cell fate or phenotype for therapeutic purposes. Thus, insight into intra- and inter-cellular crosstalk may better showcase the role of monocytes and macrophages in tumor immunity. Some of these cells hinder tumor growth and are essential in effective tumor therapies, particularly immunotherapy. Such a delicate balance argues against systemic elimination of cells using a generic cell-type marker. It should also be emphasized that myeloid cells crosstalk with each other, and in many instances when one cell-type is removed (TAMs), there may be a subsequent increase of another (TANs). These complex cellular interactions within the TME, as well as those between the acquired and innate immune systems, although incompletely understood, should be at the forefront of future investigation, as immunotherapy is undoubtedly promising. A better understanding of the mechanisms and axes controlling tumor context-specific monocytes and tissue-resident macrophage phenotypes, is essential for the rational development of methods that can favorably alter their functions in HCC.

## Figures and Tables

**Table 1:** Summary of studies evaluating the role of TAMs in HCC pathogenesis.

Study (Year)	Study Subjects	Primary Outcome	Secondary Outcome
Yang et al. (2018) [59]	Human/Animal	Wnt/β-catenin activation promotes M2 Mϕ polarization through c-Myc.	Nuclear accumulation of β-catenin is positively correlated with M2-like TAMs in human HCC biopsies.
Chen et al. (2016) [60]	Human	High level of infiltration of IL21+ TFH-like cells induces pro-tumorigenic M2b Mϕ polarization and HCC growth.	Fcγ receptor–TLR cross-talk is required for M2b Mϕ polarization and subsequent upregulation of the M2 markers IL10 and CCL1.
Zhang et al. (2016) [89]	Human	CD169+ Mϕs could suppress tumor progression by enhancing CD8+ T-cell activity in human HCC.	Tumor-induced autocrine TGF-β downregulates CD169 expression by Mϕs.
Zhang et al. (2018) [63]	Human/Animal	M1 Mϕs accumulate in the SPON2-abundant regions of HCC, exhibiting antitumor immune responses through distinct integrin-Rho GTPase-Hippo pathways.	SPON2 interactions with integrin α4β1 receptors activate RhoA and Rac1, resulting in F-Actin accumulation that promotes M1 Mϕ infiltration and migration.
Zhao et al. (2012) [66]	Human	IL-6/STAT3 signaling pathway regulates Mϕ polarization in HCC, and its inhibition suppresses tumor formation and metastases.	The TME induces the formation of suppressive MΦs, leading to early T cell activation and subsequent MΦ IDO expression in HCC.
Zhang et al. (2018) [75]	Human/Animal	M2 Mϕs under FAO-mediated upregulated secretion of IL-1β enhance the proliferation, migration and invasion of HCC cells.	IL-1β induction is reactive oxygen species-dependent and NLRP3-dependent.
Schneider et al. (2012) [77]	Animal	Chemically induced hepatocarcinogenesis triggers an intrahepatic accumulation of macrophages and cytotoxic T cells.	Activation of adaptive immunity-related pathways affect survival of patients with HCC.
Mano et al. (2013) [78]	Human/Animal	TAMs correlate with pSTAT3 expression in HCC, expressing high levels of IL-6.	IL-6 stimulates cell proliferation and the migration of human HCC cell lines.
Guo et al. (2017) [81]	Human	The expression of CD68, CD163 and CD206, the M2-TAM markers, is significantly higher in HCC tissues than in normal hepatic tissues.	IL-17 expression by M2-TAMs is augmented by oxaliplatin treatment and reduces oxaliplatin-induced apoptosis in HCC cells by activating CMA.
Bartneck et al. (2019) [90]	Animal	Pro-inflammatory CCR2+ TAMs accumulate at the highly vascularized HCC border, whereas CD163+ immune-suppressive TAMs accrue in the HCC center.	CCR2+ M2 Mϕs express CCL6, which is involved in immune cell recruitment, and NF-κB, which is associated with many inflammatory processes.
Zhang et al. (2018) [85]	Human/Animal	M2 Mϕs enhance IL-1β secretion in HCC under moderate hypoxic conditions via an HIF-1α/IL-1β signaling loop, leading to increased metastasis and the poor prognosis of HCC patients.	TLR4/TRIF/NF-κB signaling mediates cell necrotic debris–induced IL-1β production by macrophages, inducing an epithelial–mesenchymal transition in HCC cells.
Zang et al. (2018) [88]	Human/Animal	Liver inflammatory macrophages of HBV-related HCC patients produce high amounts of IL-23, which in turn augment macrophage-induced angiogenesis in the JAK-STAT3 pathway.	Blocking IL-23 cytokine activity decreased liver cancer development in the murine model.
Wang et al. (2017) [69]	Human	M2 Mϕs promote HCC progression by secreting cytokine factor CCL18.	CTGF is the key factor secreted by mesenchymal-like HCC cells that leads to the polarization of Mϕs, promoting HCC progression.

Mϕ: macrophage; TAM: tumor-associated macrophage; HCC: hepatocellular carcinoma; TME: tumor microenvironment; IL: interleukin; TFH: follicular helper T; TLR: toll-like receptor; CCL: CC chemokine ligand; CCR: CC chemokine receptor; SPON2: spondin 2; STAT: signal transducer and activator of transcription; IDO: indoleamine 2,3 dioxygenase; FAO: fatty acid oxidation; NLRP3: NOD-, LRR- and pyrin domain-containing protein 3; CMA: chaperone-mediated autophagy; NF-κB: nuclear factor kappa-light-chain-enhancer of activated B cells; HIF: hypoxia inducible factor; TRIF: toll/interleukin-1 receptor domain-containing adaptor protein inducing interferon beta; HBV: hepatitis B virus; JAK: janus kinase; CTGF: connective tissue growth factor.

**Table 2:** Summary of studies evaluating the role of TAMs in HCC prognosis.

Study (Year)	Study Subjects	Primary Outcome	Secondary Outcome
Ke et al. (2019) [97]	Human/Animal	ΜiR-148b deficiency promotes HCC growth and metastasis through CSF1/CSF1R-mediated TAM infiltration.	Decreased miR-148b levels and increased TAM infiltration were correlated with worse prognoses for HCC patients.
Chen et al. (2019) [98]	Human/Animal	The levels of PFKFB3 + CD68+ cell infiltration in peritumoral tissues were negatively correlated with the overall survival and could serve as an independent prognostic factor for survival in patients with HCC.	Tumor-derived soluble factors upregulated PFKFB3 in TAMs, which in turn mediated the increased expression of PD-L1 by the activation of the NF-kB signaling pathway.
Li et al. (2019) [99]	Human/Animal	SIRT4 is downregulated in CD68+ M2-like TAMs and correlates with the poor survival of HCC patients.	Downregulation of SIRT4 in TAMs modulates the alternative activation of macrophages and promotes HCC development via the FAO-PPARδ-STAT3 axis.
Zhang et al. (2016) [100]	Human	High peritumoral HMGB1 expression and TAM count, which correlated positively with tumor size and the BCLC stage of HCC, are independent prognostic factors for OS and RFS.	The degree of TAM infiltration is higher in peritumoral tissues with high HMGB1 expression than in peritumoral tissues with low HMGB1 expression.
Kono et al. (2016) [102]	Human	M-CSF density, CD163 index and CD31 index in peritumoral tissues are independent prognostic factors HCC patients.	M-CSF, M2 Mϕs and angiogenesis in the peritumoral liver tissue are correlated with DFS after surgery.
Ohno et al. (2014) [103]	Human/Animal	Increased intratumoral infiltration of CD204-positive or MCT4-positive macrophages suggested shorter OS in patients with HCC.	MCT4+ HCC cases correlated with higher intratumoral M2-Mϕ and higher intratumoral MCT4-positive Mϕ.
Zhu et al. (2008) [105]	Human/Animal	High peritumoral M-CSF and Mϕs are associated with HCC progression, disease recurrence and poor survival after hepatectomy.	High peritumoral M-CSF and Mϕ density correlate with large tumor size, presence of intrahepatic metastasis and advanced stage.
Zhu et al. (2014) [106]	Human	OPN, combined with PTMs, is an independent prognostic factor for both OS and TTR of early-stage HCC after curative resection.	PTM expression is closely associated with tumor recurrence and survival in HCCs with higher OPN levels, but is not significant in those with lower OPN expression.

HCC: hepatocellular carcinoma; TAM: tumor-associated macrophage; Mϕ: macrophage; CSF: colony stimulating factor; CSF1R: colony stimulating factor-1 receptor; NF-Kb: nuclear factor kappa-light-chain-enhancer of activated B cells; PD-L1: programmed death-ligand 1; SIRT4: sirtuin 4; FAO: fatty acid oxidation; STAT3: signal transducer and activator of transcription 3; PPARδ: peroxisome proliferator-activated receptor delta; HMGB1: high mobility group box 1; BCLC: Barcelona clinic liver cancer; OS: overall survival; RFS: recurrence-free survival; M-CSF: macrophage colony-stimulating factor; MCT4: monocarboxylate transporter-4; OPN: osteopontin; PTM: peritumoral macrophage; TTR: time to response.

**Table 3:** Summary of studies evaluating the role of TAMs in HCC therapy.

Study (Year)	Study Subjects	Outcome
Zhang et al. (2010) [109]	Animal	Depletion of macrophages by clodrolip or zoledronic acid, in combination with sorafenib, significantly inhibited HCC progression, angiogenesis and lung metastasis compared with the use of sorafenib alone.
Wu et al. (2019) [110]	Animal	Sorafenib, at a subpharmacologic level, augments the antitumor effects of mCAR-T cells, by promoting IL12 secretion in TAMs.
Sprinzl et al. (2013) [111]	Animal	Sorafenib triggers the proinflammatory activity of TAMs and subsequently induces antitumor NK cell responses in a cytokine- and NF-κB-dependent fashion.
Yao et al. (2017) [113]	Animal	The natural CCR2 antagonist 747 elevates the number of CD8+ T cells in HCC by blocking TAM-mediated immunosuppression and inhibiting HCC progression in a CD8+ T-cell-dependent manner.
Yang et al. (2012) [116]	Animal	E2 suppresses macrophage alternative activation and, as a result, HCC progression, by keeping ERβ away from interacting with ATP5J, thus inhibiting the JAK1-STAT6 signaling pathway.
Tsuchiyama et al. (2008) [117]	Animal	Recombinant adenovirus vector expressing MCP-1 enhances the antitumor effects of suicide gene therapy against HCC by M1 macrophage activation.
Guerra et al. (2017) [118]	Animal	Hydrogel-embedded M1 macrophages upregulate nitrite and TNF-α, activating caspase-3-induced apoptosis and HCC regression.
Xiao et al. (2015) [120]	Animal	Macrophage phagocytosis of HCC cells is increased after treatment with CD47 antibodies that block CD47 binding to SIRPα.
Tan et al. (2016) [121]	Animal	IRE1α inhibition by genipin on TAMs reduces XBP-1 splicing and NF-κB activation, suppressing HCC proliferation.
Wang et al. (2019) [122]	Animal	Co-delivery of glycyrrhizin and doxorubicin attenuates the activation of macrophages and their phagocytic activity, enhancing the therapeutic efficacy for HCC.
Sprinzl et al. (2015) [112]	Animal	Sorafenib lowers mCD163 and IGF-1 release by M2 macrophages, decelerating M2-macrophage-driven HepG2 proliferation.
Deng et al. (2016) [114]	Human/Animal	Sorafenib abolished polarized-macrophage-induced EMT and migration of HCC cells in vitro and also attenuated HGF secretion in polarized macrophages, decreasing plasma HGF in patients with HCC.
Wei et al. (2015) [115]	Animal	Sorafenib inhibited miR-101 expression, enhanced DUSP1 expression and lowered TGF-β and CD206 release in M2 cells, slowing macrophage-driven HCC.
Li et al. (2018) [123]	Animal	In mice with HCC, injection of LipC6 reduces the number of TAMs, their production of ROS and their ability to suppress the anti-tumor immune response.
Yin et al. (2014) [125]	Animal	nsPEFs enhance HCC cell phagocytosis by human macrophage cell (THP1) in vitro.
Chen et al.(2014) [124]	Animal	In vivo, low doses and multiple treatments of nsPEF significantly elevate macrophage infiltration in HCC tumors, contributing to tumor ablation.

TAM: tumor-associated macrophage; HCC: hepatocellular carcinoma; TME: tumor microenvironment; IL: interleukin; mCAR: mouse chimeric antigen receptor; NK: natural killer; NF-kB: nuclear factor kappa-light-chain-enhancer of activated B cells; CCR2: C-C chemokine receptor type 2; E2: estradiol; ERβ: estrogen receptor beta; ATP5J: ATP synthase-coupling factor 6; JAK1: janus kinase 1; STAT6: signal transducer and activator of transcription 6; MCP: monocyte chemoattractant protein; TNF-α: tumor necrosis factor alpha; SIRPα: signal regulatory protein alpha; IRE1α: inositol-requiring endoribonuclease 1α; XBP-1: x-box-binding protein 1; IGF-1: insulin-like growth factor-1; EMT: epithelial–mesenchymal transition; HGF: hepatocyte growth factor; DUSP1: dual specificity phosphatase 1; TGF-β: transforming growth factor beta; LipC6: nanoliposome-loaded C6- ceramide; ROS: reactive oxygen species; nsPEF: nanosecond pulsed electric field.
